# TET2单核苷酸多态性位点I1762V在急性髓系白血病患者中的临床及预后意义

**DOI:** 10.3760/cma.j.issn.0253-2727.2022.03.010

**Published:** 2022-03

**Authors:** 扬威 李, 珍 郭, 琳琳 王, 玲 周, 晓东 吕, 永平 宋

**Affiliations:** 1 郑州大学附属肿瘤医院、河南省肿瘤医院中心实验室，郑州 450008 Central Lab, the Affiliated Cancer Hospital of Zhengzhou University, Henan Cancer Hospital, Zhengzhou 450008, China; 2 郑州大学附属肿瘤医院、河南省肿瘤医院血液科，郑州 450008 Department of Hematology, the Affiliated Cancer Hospital of Zhengzhou University, Henan Cancer Hospital, Zhengzhou, Henan 450008, China

**Keywords:** 基因，TET2, 单核苷酸多态性, 基因突变, 白血病，髓系，急性, Gene, TET2, Single nucleotide polymorphism, Gene mutation, Leukemia, myeloid, acute

## Abstract

**目的:**

探讨TET2单核苷酸多态性（SNP）位点I1762V在急性髓系白血病（AML）患者中的临床意义及其对预后的影响。

**方法:**

采用高通量测序方法，对2016年7月至2019年12月于河南省肿瘤医院血液科就诊的413例AML患者骨髓样本中的58种血液肿瘤相关基因进行靶向测序。统计TET2 SNP位点I1762V的检出情况，并分析其与患者的临床特征、体细胞突变、预后的相关性。

**结果:**

154例AML患者检出TET2 SNP位点I1762V，检出比例为37.3％，与公共数据库（NyuWa Chinese Population Variant Database，NCVD）对照人群相比差异有统计学意义（*χ*^2^＝72.4，*P*<0.001）；TET2 SNP位点I1762V与AML患者的性别、年龄、染色体核型等均无明显相关性（*P*值均>0.05）。携带TET2 SNP位点I1762V的患者伴发NPM1基因突变和KIT基因突变的比例显著高于非携带者（*P*<0.001），且NPM1和KIT突变互斥发生。生存分析结果显示，携带TET2 SNP位点I1762V的AML患者总生存（OS）率、无进展生存（PFS）率显著高于野生型患者（*HR*＝0.57，*P*＝0.030；*HR*＝0.55，*P*＝0.020）；不论是否携带TET2 SNP位点I1762V，DNMT3A突变的AML患者OS率、PFS率均低于野生型患者（*HR*＝1.79，*P*＝0.030；*HR*＝1.74，*P*＝0.040）。

**结论:**

TET2 SNP位点I1762V可能与AML相关，可用于指导治疗和预后评估。TET2 SNP位点I1762V是影响AML患者预后的有利因素。

## Abstract (en)

**Objective:**

This study aimed to investigate the clinical and prognostic significance of TET2 single nucleotide polymorphism I1762V in patients with acute myeloid leukemia（AML）.

**Methods:**

The high-throughput sequencing method was used to sequence 58 hematological tumor-related genes in bone marrow samples from 413 patients with AML. TET2 I1762V and other somatic mutations were annotated and compared with patients' clinical information and prognosis.

**Results:**

I1762V was found in 154 patients with AML, which was significantly different from the general population in NyuWa Chinese Population Variant Database（*χ*2＝72.4, *P*<0.001）. I1762V was not related to sex, age, and karyotype of patients with AML（*P*>0.05）. Patients with I1762V had a significantly higher proportion of NPM1 and KIT gene mutations than others（*P*<0.001）. NPM1 and KIT mutations were mutually exclusive. The survival analysis results revealed that the overall survival（OS）and progression-free survival（PFS）of patients with AML with I1762V were significantly greater than those of wild-type patients（*HR*＝0.57, *P*＝0.030; *HR*＝0.55, *P*＝0.020）, whereas the OS and PFS in patients with AML with DNMT3A mutation（with or without I1762V mutation）were lower than those of wild-type patients（*HR*＝1.79, *P*＝0.030; *HR*＝1.74, *P*＝0.040）.

**Conclusion:**

TET2 SNP I1762V has been linked to AML. I1762V is a prognostic factor of patients with AML, which can be used to guide the treatment and evaluate the prognosis of AML.

急性髓系白血病（AML）是一种起源于骨髓髓系造血干/祖细胞的恶性肿瘤。目前公认的AML发病原因是各类理化因素如苯、杀虫剂、电离辐射、化疗药物等诱导的髓系造血干/祖细胞染色体异常或基因突变[1]。随着高通量测序技术的发展，多种获得性体细胞突变被用于AML的诊断、治疗和预后评估[2]。同时，许多单核苷酸多态性（SNP）导致的遗传易感性也被认为是AML发生的重要原因[3]。TET2基因编码一种甲基胞嘧啶双加氧酶，催化甲基胞嘧啶转化为5-羟甲基胞嘧啶，有助于骨髓生成的表观遗传调节，TET2基因突变会导致甲基化失调和髓样转化[4]。TET2 SNP位点I1762V（rs2454206^AG/GG^，c.5284A>G, p.Ile1762Val）在中国人群中等位基因频率约为21％，有研究表明携带该位点突变的儿童AML患者预后较好[5]–[6]。本研究通过对413例AML患者的高通量测序结果进行综合分析，探讨TET2 SNP位点I1762V在AML患者中的临床意义及其对预后的影响。

## 病例与方法

1. 病例资料：研究对象为2016年7月至2019年12月于河南省肿瘤医院血液科就诊的413例AML患者，包括初诊AML患者378例、继发AML患者13例和复发AML患者22例。患者中男227例，女186例，中位年龄为46（2～89）岁。AML的诊断和分型符合《成人急性髓系白血病（非急性早幼粒细胞白血病）中国诊疗指南（2017年版）》[7]。另外随机选取234例非髓系血液肿瘤患者，包括急性淋巴细胞白血病148例、慢性淋巴细胞白血病68例和非血液肿瘤18例，中位年龄为41（2～77）岁，同样检测其TET2 SNP位点I1762V发生情况。补充对照组选择公共数据库（NyuWa Chinese Population Variant Database，NCVD）中的2999名中国人群数据。

2. 样本收集及处理：采集患者骨髓2～3 ml，EDTA或枸橼酸钠抗凝，使用血液基因组DNA提取试剂盒（北京天根生化科技有限公司产品）提取基因组DNA，经NanoDrop 2000定量后保存或用于后续检测。

3. 扩增子法基因突变检测：使用自主设计，美国Thermofisher公司合成的扩增子panel（58种AML相关基因热点区域）进行文库构建，对照组使用的扩增子panel覆盖TET2基因，文库构建完成后在Ion Torrent PGM测序平台上进行测序。下机数据使用Torrent Suite进行处理分析、Ion reporter进行突变位点注释，使用IGV软件对注释出的突变位点进行人工检查，使用Sanger测序对疑似假阳性位点进行确认。最终单样本测序质控满足panel覆盖率≥95％，平均测序深度≥1000×，体细胞突变检测灵敏度为5％。

4. 染色体核型分析：染色体核型分析采用R显带技术，根据《人类细胞遗传学国际命名体制（ISCN2013）》进行描述。

5. 治疗：治疗方案参照成人AML中国诊疗指南2011、2017年版，依据患者年龄、危险度预后分层、依从性等进行综合评价。12例急性早幼粒细胞白血病（APL）患者采用全反式维甲酸（ATRA）、ATRA联合化疗和（或）亚砷酸（ATO）方案的诱导治疗，缓解后采用ATRA联合化疗和（或）ATO方案巩固治疗及维持治疗。非APL的AML患者进行包含蒽环/蒽醌类、阿糖胞苷和（或）高三尖杉酯碱（HHT）的联合诱导化疗，其中接受标准剂量方案290例，低强度化疗方案31例；缓解后采用中高剂量阿糖胞苷或标准“3+7”方案巩固化疗；有80例患者行异基因造血干细胞移植。

6. 随访：主要通过电话或查阅门诊复查记录进行随访，随访截至2021年7月。中位随访时间为28（2～60）个月，主要评价总生存（OS）期和无进展生存（PFS）期。OS期指自确诊之日至死亡或随访截止的时间；PFS指自确诊之日至疾病复发、进展或死亡的时间。

7. 统计学处理：统计学分析采用R软件。计数资料的比较采用卡方检验或Fisher精确检验；计量资料的比较使用*t*检验或多因素方差分析；使用Kaplan-Meier法分析计算不同组别的OS期和PFS期，并用对数秩（Log-rank）检验比较组间差异[8]。采用Cox比例风险回归模型进行多因素分析[9]。*P*<0.05为差异有统计学意义。

## 结果

1. TET2 SNP位点I1762V的检出情况及与AML患者临床特征相关性分析：高通量测序结果显示，413例AML患者中有154例检出TET2 SNP位点I1762V，检出比例为37.3％，突变型G等位基因频率为35.0％（等位基因频率统计方式为纯合型×2+杂合型）。为验证对照组与AML组群体性关系，额外统计5个SNP位点（TP53 rs1042522、ATRX rs3088074、SETBP1 rs3744825、TET2 rs12498609、SETBP1 rs1064204）进行等位基因频率比较，5个SNP位点在AML组与两个对照组中的等位基因频率均符合哈温平衡（HWE>0.05），可认定对照组与AML组属于同一遗传群体，能够进行比较分析。

AML组患者与公共数据库（NyuWa Chinese Population Variant Database，NCVD）中的中国人群突变型G等位基因频率（21.6％）相比差异有统计学意义（*χ*^2^＝72.3，*P*<0.001）；同时，234例非髓系血液肿瘤患者中突变型G等位基因频率为21.0％，与中国人群G等位基因频率相比差异无统计学意义（*P*>0.05），与AML患者G等位基因频率相比差异有统计学意义（*χ*^2^＝29.5，*P*<0.001）。

413例AML患者中70例未见染色体核型结果，检出正常核型189例，t（8;21）44例，t（15;17）12例，复杂核型19例，其他核型78例。TET2 SNP位点I1762V与AML患者临床特征相关性分析结果见表1，I1762V与AML患者的性别、年龄、危险度分层、疾病状态（初诊/复发/继发）及核型均无显著相关性（*P*值均>0.05）。

**表1:** TET2单核苷酸多态性位点I1762V与急性髓系白血病（AML）患者临床特征的相关性［例（％）］

临床特征	突变型（154例）	野生型（259例）	*χ*^2^值	*P*值
性别			0.718	0.397
男	80（35.2）	147（64.8）		
女	74（39.8）	112（60.2）		
年龄（岁）			3.260	0.515
1～<18	13（32.5）	27（67.5）		
18～<30	26（38.2）	42（61.8）		
30～<46	36（40.0）	54（60.0）		
46～<60	42（42.4）	57（57.6）		
≥60	37（31.9）	79（68.1）		
危险度分层			0.406	0.939
低危组	17（41.5）	24（58.5）		
中危组	58（36.7）	100（63.3）		
高危组	46（36.2）	81（63.8）		
不详	33（37.9）	54（62.1）		
核型				0.337
正常	79（41.8）	110（58.2）		
t（8;21）	18（40.9）	26（59.1）		
t（15;17）	3（25.0）	9（75.0）		
复杂核型	8（40.0）	12（60.0）		
其他异常	23（29.5）	55（70.5）		
疾病状态				0.898
初诊	137（36.2）	241（63.8）		
复发	9（40.9）	13（59.1）		
继发	5（38.5）	8（61.5）		

2. TET2 SNP位点I1762V与AML患者体细胞突变相关性分析：413例AML患者中有345例检出体细胞突变，检出的突变基因38种，按照基因功能分为5类：剪切因子、表观遗传、细胞增殖和凋亡、信号传导和转录调节。将体细胞突变的AML患者按照是否携带TET2 SNP位点I1762V分为两组，突变型组126例、野生型组219例（图1）。突变型组与野生型组5类基因功能分类的突变情况差异无统计学意义（*P*＝0.770）。

**图1:**
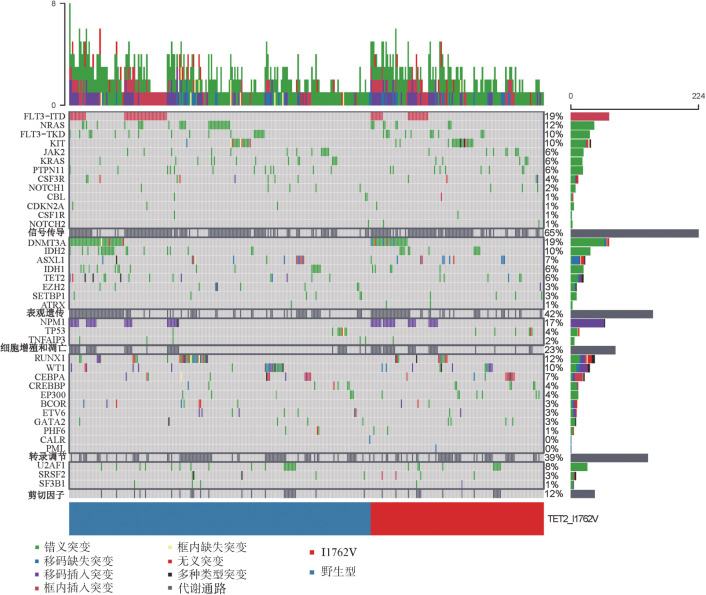
急性髓系白血病患者体细胞突变与TET2 单核苷酸多态性位点I1762V相关性分析

基因功能相关性分析结果显示，野生型组DNMT3A体细胞突变与TET2体细胞突变、IDH2体细胞突变相关联（TET2：双野生型170例/双突变型7例/TET2单突变型9例/DNMT3A单突变型33例，*P*<0.05；IDH2：171/14/8/26，*P*<0.001），突变型组DNMT3A突变与TET2体细胞突变、IDH2突变无显著关联（TET2：91/4/8/23，*P*>0.05；IDH2：93/0/6/27，*P*>0.05）。在伴发的基因突变中，突变型组伴发NPM1和KIT基因突变的比例显著高于野生型组（*P*<0.05）（图2），I1762V与TET2体细胞突变的发生无明显相关性（I1762V：6/126，野生型：19/219，*P*＝0.260）。

**图2:**
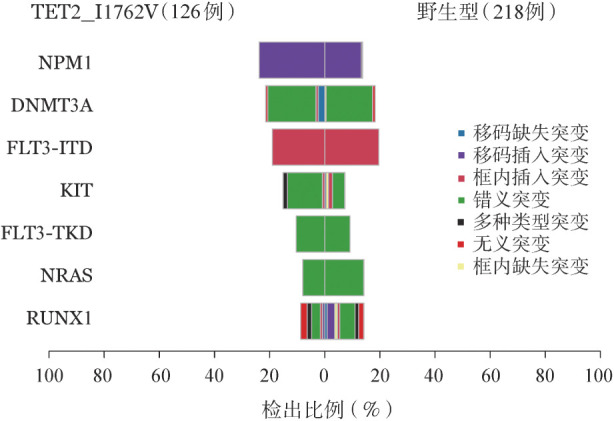
急性髓系白血病患者TET2单核苷酸多态性位点I1762V伴发体细胞突变分析

3. TET2 SNP位点I1762V与初诊AML患者预后的相关性分析：378例初诊患者中25例失访，由于APL患者及异基因造血干细胞移植患者的治疗方案及良好预后的特殊性，排除上述患者，将261例未进行造血干细胞移植且非APL患者纳入预后分析。依据患者随访记录，将AML患者分别按照年龄、性别、TET2 SNP位点I1762V突变、单一体细胞突变以及突变两两组合进行分组，比较不同组别的OS率与PFS率，筛选与AML患者预后相关的因素。单因素预后分析结果显示，I1762V组OS率、PFS率显著高于对照组。DNMT3A突变组、DNMT3A突变伴NPM1突变组患者OS率、PFS率均显著低于对照组（表2）。为排除NPM1突变预后良好对分析的干扰，我们将样本按照I1762V^+^NPM1^−^组、NPM1^+^组和I1762V^−^NPM1^−^组进行预后分析，I1762V^+^NPM1^−^组患者OS率、PFS率显著高于其他两组（OS：*HR*＝0.68，*P*<0.05；PFS：*HR*＝0.70，*P*<0.05）（图3）。

**表2:** 影响急性髓系白血病患者预后的单因素分析

变量	例数（突变型/野生型）	总生存		无进展生存	
		*HR*（95％*CI*）	*P*值	*HR*（95％*CI*）	*P*值
年龄	261	1.01（1.00, 1.02）	0.09	1.01（1.00～1.02）	0.12
性别	261	0.97（0.61～1.53）	0.88	0.96（0.61～1.52）	0.86
TET2 SNP位点I1762V	104/157	0.57（0.34～0.93）	0.03	0.55（0.34～0.91）	0.02
DNMT3A突变	42/219	1.79（1.05～3.05）	0.03	1.74（1.02～2.96）	0.04
DNMT3A合并NPM1突变	23/238	2.13（1.14～3.95）	0.02	2.03（1.09～3.78）	0.02

注：SNP：单核苷酸多态性

**图3:**
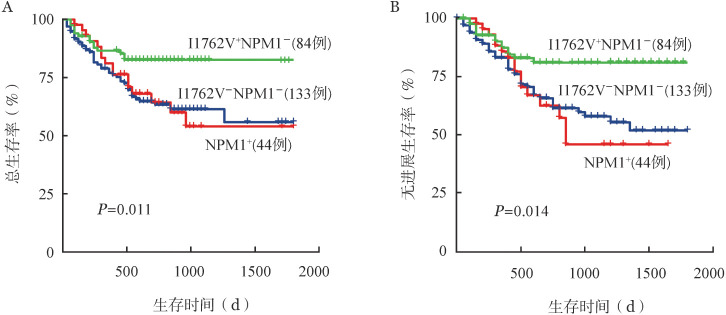
TET2单核苷酸多态性位点I1762V对急性髓系白血病患者总生存（A）和无进展生存（B）的影响（排除NPM1突变干扰）

## 讨论

基因突变在AML的诊断、分类、治疗和预后评估中起着关键作用，AML的发生和进展与调控各类功能的基因突变相关，包括促进细胞增殖和凋亡、表观遗传调节基因等[1]。长久以来，体细胞突变通常被认为是肿瘤发生的驱动因子，而SNP位点在肿瘤发生发展过程中的作用往往被忽视[10]。事实上，许多SNP位点已被证明是肿瘤发生发展的重要驱动因子，与体细胞突变不同，SNP位点可能通过改变RNA稳定性、RNA折叠和剪接的变化、tRNA选择的差异以及非编码RNA的结合能力等影响蛋白的表达，进而影响蛋白功能的发挥[11]–[12]。TET2蛋白在造血过程中发挥着重要的作用，包括调控造血干细胞的自我更新、分化和单核细胞的末端分化，TET2基因突变可能导致甲基化失调和髓系转化[13]。近年来，TET2 SNP位点在血液肿瘤中的影响也有陆续报道：TET2 rs3733609C/T突变被发现是骨髓增殖性肿瘤发病的遗传易感因素；K1500fs、K1363fs等截短型突变在家族性血液肿瘤患者中检出；rs2454206^AG/GG^（I1762V）SNP位点是儿童AML的预后良好指标，其在成人AML中的作用尚存争议，可能存在种族差异[14]–[16]。本研究通过对413例AML患者的临床信息、高通量测序结果、染色体核型等进行综合分析，发现TET2 SNP位点I1762V与AML患者的体细胞突变情况及预后密切相关。

本研究中AML患者I1762V突变型G等位基因频率为35.0％，显著高于234例对照人群（21.0％）和公共数据库中中国人群的G等位基因突变频率（21.6％），证明I1762V可能与AML有关。进一步比较发现，I1762V与患者的年龄、性别、核型等临床信息无显著相关性，表明I1762V并非单独的AML遗传易感因素，其影响AML发生的机制尚待进一步研究。

TET2、DNMT3A等表观遗传调控基因突变导致的单倍体不足可能并不直接触发细胞癌变，往往需要额外的细胞遗传学改变如体细胞突变来诱导疾病的发生，这也解释了为什么大多数AML患者都有一个以上的基因突变[17]–[18]。本研究对携带TET2 SNP位点I1762V的AML患者伴发体细胞突变情况进行了比较分析。与对照组相比，I1762V组的体细胞突变伴发或互斥情况有所不同：在携带I1762V突变的AML患者中，DNMT3A与TET2基因体细胞突变、IDH2基因突变的关联伴发并不显著，提示I1762V可能与DNMT3A基因突变导致的功能失调类似。研究还发现，携带I1762V的AML患者伴发NPM1或KIT基因突变的比例高出对照组一倍，提示I1762V与NPM1、KIT基因突变的发生显著相关。

AML的预后受多个因素的影响，单个突变的预后意义通常因其他突变的存在而改变。例如具有双等位基因CEBPA突变的AML单独发生时预后良好，伴FLT3突变或细胞遗传学异常发生时预后较差，NPM1与FLT3-ITD突变的共同出现会导致不良预后的发生[19]。本研究对AML患者的预后影响因素进行了综合分析，筛选出预测AML患者预后的关键分子组合：携带TET2 SNP位点I1762V的AML患者OS率、PFS率显著高于未携带者，而DNMT3A突变伴发NPM1突变的AML患者预后较差。研究结果提示TET2 SNP位点I1762V可作为AML患者的预后良好因素，DNMT3A突变伴发NPM1突变是AML患者的预后不良因素。本研究证明了TET2 SNP位点I1762V可能与AML的发生有关，且I1762V与NPM1、KIT基因突变的发生有一定相关性，但具体影响因素有待进一步研究。受限于样本数量，本研究未对AML疾病进行分类讨论分析，下一步需扩大样本量进行更深层次的研究。

综上所述，TET2 SNP位点I1762V在AML患者中的检出率高于正常人群；I1762V影响了AML患者体细胞突变的伴发或互斥；I1762V与AML患者的预后密切相关。TET2 SNP位点I1762V对AML患者的诊断、治疗和预后评估有重要的指导意义。当前对于SNP位点的研究仍受限于检测群体和数据的积累，随着高通量测序技术在临床检测中应用的不断完善，对SNP位点的深入研究能够使我们更好地预测肿瘤的发生、发展，为个性化的精准治疗策略提供指导。
